# You Talking to Me? Quorum Sensing in Symbiotic Microbes and Their Response to Environmental Variation

**DOI:** 10.3390/microorganisms14091876

**Published:** 2026-08-24

**Authors:** Pedro Antonio Pérez-Ferrer, Michele Kiyoko Nishiguchi

**Affiliations:** Molecular and Cell Biology Department, School of Natural Sciences, University of California Merced, Merced, CA 95343, USA; pperez40@ucmerced.edu

**Keywords:** quorum sensing, autoinducers, host–microbe symbiosis, environmental modulation, signal stability, bacterial communication, pH, temperature

## Abstract

Bacterial communication or quorum sensing (QS) is a common yet complex system where multiple factors influence the extent to how this chemical dialog is transferred from a single clone to the larger community of microbes in the population. More often, when bacteria are in large concentrations, their genetic and subsequent biochemical response to different chemical cues is influenced by not only which microorganisms are present but also the environmental variables that surround those individuals. This is especially relevant when symbiotic bacteria are dependent upon host functions yet are in high enough concentrations that can manifest their own behaviors in response to the present host prior, during, and after colonization. This review will examine the various abiotic and biotic factors that regulate QS when bacteria are in the process of detecting, colonizing, and persisting in a host that uptakes its microbial partner from the environment, as well as the consequences of multiple stressors on this dynamic communication process.

## 1. Introduction

Bacteria rarely act as independent units; rather, they interact with the environment and with individuals of their own and other species. Bacterial consortia or communities generally work together and require a mechanism for signals to pass from one individual to another to coordinate collective actions. Examples such as activating bioluminescence, producing biofilm, excreting virulence factors, and switching metabolic programs are often critical in host-associated settings and can only occur when the concentration of bacterial cells reaches a specific threshold and the population is at optimal conditions to ensure success [1,2]. To coordinate these behaviors, many bacteria rely on quorum sensing (QS), defined as a circuit in which bacteria produce small signaling molecules, called autoinducers, that accumulate in the environment and, once they reach a certain concentration, are detected by specific receptors. This interaction between the signal and the receptor triggers coordinated changes in gene expression across the population [3,4]. This is often presented as the most straightforward, original definition of QS.

**Figure 1:**
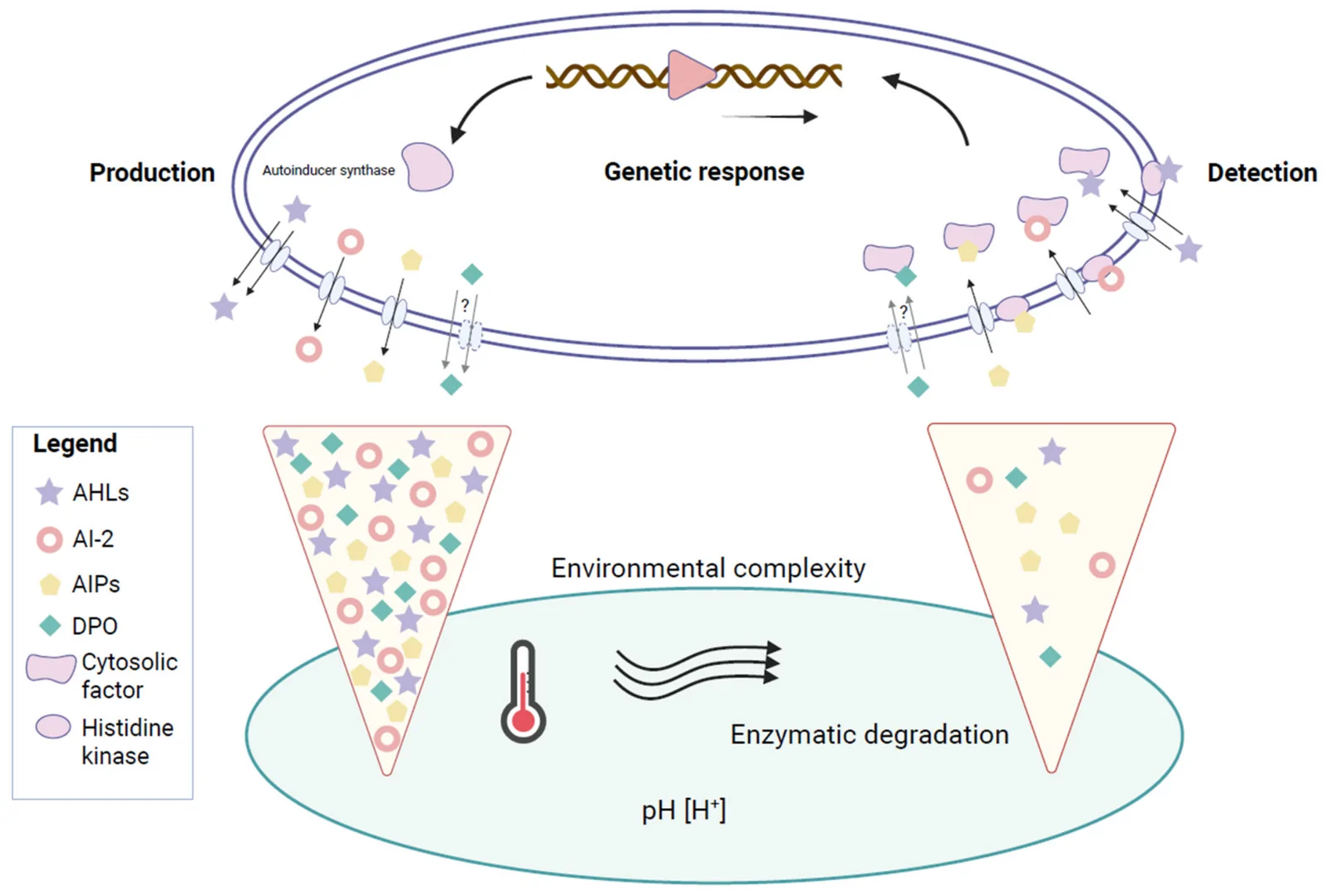
Cellular architecture and environmental constraints of bacterial communication. A generalized model showing the lifecycle of key quorum-sensing signals from production to detection. The figure distinguishes signals by their transport methods, ranging from active transmembrane transport (AI-2, AIPs) and mixed transport (AHLs) to diffusion with uncharacterized mechanisms (in the case of DPO represented with dashed channels). Target receptors include both surface-bound histidine kinases and cytosolic transcription factors. The bottom panel visually depicts the attenuation of the extracellular signal pool driven by environmental factors (pH, temperature, enzymatic degradation) before the signal interacts with the receptor.

However, as research has progressed, the traditional view that autoinducer accumulation is simply a proxy for how many cells are nearby has been redefined. QS is notably a dynamic process, where both the signaling molecules and the receptors can be strongly influenced by environmental factors [5] (Figure 1). Importantly, the concentration of a signal reflects not only how much is being produced but also how the environment influences that molecule once it is released. However, because signal accumulation also depends on how quickly molecules diffuse, degrade, or are swept away, QS can also be explained by a phenomenon known as diffusion sensing, where cells are effectively testing whether autoinducers will remain local or be lost in the environment [6,7,8]. Finally, efficiency sensing integrates these ideas by emphasizing that the benefit of initiating a collective response depends on the balance between cell density and the physicochemical context of the environment, which determines signal retention and its integration with the receptor and the response [9,10].

Signals, moreover, must be detected once they are released into the surrounding environment. This process involves receptors such as transcription factors, membrane-bound histidine kinases, or orphan receptors, which do not behave passively but are dynamic proteins whose activity changes depending on the environment [11]. Temperature, pH and ionic strength, membrane state, and the local chemical environment (the presence of compatible ligands [12], antagonists [13], degradation products, and host-derived mimics [14,15]) can change folding, binding, or signaling output, such that the same external autoinducer concentration can lead to different transcriptional responses in variable environments [16] (Figure 1).

In this review, we focus on how the environment can affect both the signal as well as its receptor and the response that is triggered by their interactions. We will present a QS view focused on symbiosis, asking how these environmentally induced variations lead to shifts in microbial behavior and its implications for the host in events such as colonization, biofilm formation, persistence, nutrient exchange, immunological modulation, and, in pathogenic contexts, the timing and intensity of virulence factors. The novelty of this review lies in presenting QS as a fundamentally dynamic process, reinforcing the idea that bacterial communication is subject to continuous environmental modification, at both the signal and receptor level and at every step of the process.

## 2. Chemical Stability and Environmental Effects on Autoinducers

Autoinducer molecules are often described as the “message” in the language of QS. However, they are fundamentally chemical entities that only successfully relay information once they are received and interpreted by a receptor. By the time this happens, the autoinducer molecule has already been exposed to the environment, and the environment has modulated what portion of the signal remains intact, how long it is available, and where it concentrates to a greater or lesser extent. In different habitats such as under variable flow, within mucus or biofilms, or across different host microenvironments, the concentration of the autoinducer molecule reflects not only how much cells produce but also their transport, retention, and the chemical environment, which can change the meaning of the same signal in different contexts [17,18].

Autoinduction was first described in luminous bacteria of the genus *Vibrio* (*Photobacterium* at the time), where it was described how the phenomenon of bioluminescence remains silent at low cell density and turns on as cells accumulate, implying that the bacterial population was responding to a diffusible cue that accumulates in the surrounding medium [1]. The next step was to chemically identify the cue, the small diffusible *N*-acyl homoserine lactone (AHL), which placed QS in a particular molecular framework (Table 1). This revealed that the signaling molecule was not a “vague” metabolite but a defined, characterizable compound [19]. All AHLs share a common chemical structure, a homoserine lactone ring linked by an amide bond to an acyl (fatty acid) side chain. Small modifications around that structure generate a surprisingly diverse chemical vocabulary. The acyl chain can vary in length, from very short ones with only two carbons in their side chain, C2-HSL [20], to long fatty acid chain AHLs, up to 20 carbons, C20-HSL [21], as well as saturations and unsaturated AHLs (e.g., C18:1-HSL in *Rhodobacteraceae* [22,23] and C14:1-HSL in marine vibrios [24]). Different substitutions in the third carbon of the fatty acid chain (e.g., 3-oxo-C12-HSL in mucopurulent respiratory secretions from cystic fibrosis patients [25] and 3-OH-C6-HSL in growth-promoting rhizobacteria [26]) can also change the way the AHL is received to other bacteria (Table 1).

While AHLs are only found in Gram-negative bacteria, there is another family of autoinducers that was discovered, later known as Autoinducer-2 (AI-2). Molecules belonging to this family are produced by both Gram-positive and Gram-negative bacteria. AI-2s were initially described in the marine bacterium *Vibrio harveyi*, where researchers observed that the supernatant of *V. harveyi* defective in the gene *luxM* and responsible for the production of AI-1 (AHL) was still capable of inducing bioluminescence in reporter cultures, predicting that a second autoinducing molecule had to be involved [33]. Chemically, AI-2, which is also known as furanosyl borate diester, is completely different from AHLs. This autoinducing molecule forms its own family [34], where the precursor 4,5-dihydroxy-2,3-pentanedione (DPD) is synthetized by the enzyme LuxS [35,36,37]. Because DPD is a very reactive molecule, it naturally closes into a ring to create furan derivatives. These derivatives work as direct precursors, which are later modified in the extracellular medium to form mature AI-2 autoinducers [38,39]. Interestingly, AI-2 can also be produced by Gram-positive bacteria, being considered a universal communication signal among bacteria [40,41]. Recent studies have pointed out its critical importance in the communication network within human microbiota [42] and in the rumen [43]. This autoinducer is emerging as a critical factor in probiotic applications. For example, AI-2 signaling promotes mucosal adhesion and colonization by probiotic strains of *Lactobacillus rhamnosus*, which accelerates the restoration of the intestinal barrier function after a dysbiosis event [44]. On the other hand, in pathogenic contexts, the AI-2 system plays a fundamental role in what is now recognized as a ‘triple threat’ in bacterial pathogenesis: the coordination between biofilm formation, antimicrobial resistance, and QS [45]. In this way, just as AI-2 can be used to promote intestinal health, it can also favor the emergence of antibiotic-resistant pathogens. The horizontal transfer of these resistance genes, which are normally plasmid-encoded, is enhanced by the biofilm matrix. Therefore, the implementation of emerging therapies that target autoinducers must be done with caution.

There is also another group of autoinducing molecules produced exclusively by Gram-positive bacteria, known as autoinducing peptides (AIPs) [46]. AIPs are a class of cyclic thiodepsipeptide QS molecules synthesized by the accessory gene regulator (*agr*) locus in Gram-positive bacteria, and they are crucial in *S. aureus* for triggering the extracellular cytotoxicity QS regulated response. The *agr* QS model comprises four components: AgrD, which is the precursor peptide; AgrB, in charge of processing and secreting AIP; AgrC as the sensor kinase that detects it; and AgrA as the response regulator [47]. In recent years, the importance of these AIPs has been frequently highlighted, especially in pathogenic states, underscoring their role as potential alternative treatments to traditional antibiotics in the fight against multidrug-resistant bacteria [48], either as weapons [49] or as targets [50].

In recent years, other strategies have emerged to silence QS in pathogenic bacteria, reducing their capacity to produce virulence factors. It is important to note that these compounds are not designed to kill the bacteria, so their use would help fight against the antimicrobial resistance derived from the use of broad-spectrum antimicrobials, which increases the selective pressure that favors the fixation of antimicrobial resistance genes in the bacterial population [51]. These strategies, encompassed by the term ‘QS Inhibition’ (QSI), include mechanisms such as inhibiting signal production, enzymatic degradation of the signal, competition for or blocking of the receptor, and even scavenging of autoinducer molecules using antibodies and macromolecules such as cyclodextrins [52]. Numerous in vitro studies have already demonstrated the promising advantages of searching for compounds with QSI capacity in diverse sources, such as marine environments [53]; the use of polyphenols, including curcumin, eugenol, and various flavonoids [54]; or even the repurposing of previously known drugs [55].

### 2.1. pH

pH is one of the environmental factors that most affects signaling through AHLs, interfering with the stability of the lactone ring, which is the most chemically fragile part of these molecules. Under alkaline conditions, AHLs undergo lactonolysis, or opening of the lactone ring, which reduces the pool of signal capable of interacting with the receptor [56]. Importantly, the rate of this process depends on temperature and on acyl chain length (short-chain AHLs tend to turn over more extensively) [57]. As a result, the same AHL can have different lifetimes across microhabitats. For example, in insect larval guts, where the environment is strongly alkaline, AHL persistence is greatly reduced. In this particular system, enzymes such as *N*-acylamino acid hydrolase (AAH), produced by bacteria inhabiting the gut, further degrade AHLs after lactonolysis, as a way to compete for nutrients in this environment with the rest of the bacterial community [58]. Meanwhile, host-associated microenvironments that are more strongly buffered or locally acidic can favor persistence of the lactone form and extend the time window in which the molecule can interact with its receptor. For example, in the *Euprymna scolopes*–*Vibrio fischeri* symbiosis, an acidic microenvironment promotes the early recruitment of *Vibrio* cells at the ciliated epithelium of the nascent light organ [59]. Once colonized, acidification of the light organ during the night facilitates light production [60]. In this system, where formation of biofilm and light production relies heavily in AHL-mediated QS, the stability of the signal is crucial for the success of the symbiont and the host.

The same dependence on signal stability appears in other host-associated systems. Coral-associated bacteria represent a taxonomically diverse microbial community [61,62], and AHL production has been widely documented in isolates recovered from the coral surface mucopolysaccharide layer (SML). This suggests that AHL-mediated signaling is an active component of the coral surface microbiome. Furthermore, the chemical identity of these signals has been verified using bacterial reporters and Liquid Chromatography–Mass Spectrometry (LC-MS). For instance, structurally confirmed *N*-(3-hydroxydecanoyl)-L-homoserine lactone (3-OH-C10-HSL) were isolated from *Vibrios* from the coral mucus [63], while isolates from Black Band Disease (BBD) mats produced C6-HSL and 3-oxo-C4-HSL [64] (Table 1). Since the SML provides a mild acidic microenvironment relative to the surrounding alkaline seawater, the lactone ring of AHLs is chemically protected against hydrolysis (lactonolysis), which effectively extends the signal’s half-life. Highlighting the biological consequence of this stability, previous research demonstrated that exposure to an AHL cocktail extracted from 11 opportunistic pathogens (including *Vibrio* and *Acinetobacter* species) was sufficient to induce significant bleaching, even in the absence of the pathogens themselves [65]. These exogenous signals destabilized the native microbiome, triggering a dysbiosis state that favored the growth of opportunistic bacteria.

Other examples of pH modulation of the QS signal include pathogenic relationships in plants and humans. In the plant pathogen *Pectobacterium carotovorum*, which causes soft-rot disease in potato, a “communication paradox” arises: the same environmental change that the bacteria must induce to cause disease also degrades the signals they rely on to coordinate that behavior. To colonize the host, these bacteria must alkalinize the plant tissue to a pH above 8 by locally raising ammonium ion concentrations, which activates their pectate lyases; yet this same alkaline shift accelerates the lactonolysis of their own AHL signals [66], shortening signal lifetime precisely when coordination is most needed. On the other hand, *Pseudomonas aeruginosa*, which is a recurrent causal agent of lung infections in the lungs of cystic fibrosis (CF) patients, thrives in opposite chemical environments. The loss of cystic fibrosis transmembrane conductance regulator (CFTR) function produces the acidification of the airway surface liquid (ASL) to a pH around 6.5 [67]. This acid environment promotes two advantages for successful QS communication. First, it chemically protects the lactonic ring of the primary AHL, 3-oxo-C12-HSL, which drives the LasR-mediated hierarchy in *P. aeruginosa* [68], from lactonolysis, extending its half-life in comparison to neutral or alkaline conditions. Second, this acidic pH favored an increase in ASL viscosity, possibly by reducing the electrostatic interactions between mucins. This hyper-viscous mucus, besides preventing correct clearance in CF patients, could trap 3-oxo-C12-HSL in the vicinity of the biofilm, avoiding its diffusion and reducing the population threshold required to trigger the QS response [69].

### 2.2. Temperature

Closely coupled with pH, temperature acts as a thermodynamic regulator of the QS signal’s half-life. Increasing temperature from 22 °C to mammalian body temperature (37 °C) significantly accelerates the hydrolysis of the AHL lactone ring [57]. Short-chain AHLs are particularly sensitive to this thermal degradation, as specifically observed in *Yersinia pseudotuberculosis* and *P. aeruginosa*. Thus, the effective range of QS is fundamentally constrained by the temperature of its surrounding ecosystem.

In the *Euprymna scolopes–Vibrio fischeri* system, the symbiont population in the light organ can communicate across segregated colonization sites via AHL-mediated signaling within the host [70], with QS playing a fundamental role in maintaining the bacterial population within the host, since dimmer strains would be eliminated for failing to contribute to bioluminescence production [71,72]. It has also been shown that temperature can influence the distribution of species within the light organ of *Sepiola* [73], since this cephalopod genus is capable of maintaining a dual-species bacterial population within the light organ, partitioning the niche between the mesophilic *V. fischeri* and the distinctly cold-adapted (psychrotrophic) species, *V. logei* [74]. While this specific question has received limited direct attention, the available evidence indicates that hosting one species adapted to colder waters and another that tolerates warmer waters within the light organ could be related to the presence of *Sepiola* both in the cold waters of the Atlantic [75,76,77] and in the warmer waters of the Mediterranean [78]. In this context, it is worth asking how two different *Vibrio* species can coexist within such a specific ecological niche. It is possible that QS plays a key role in the persistence of both species by allowing coordinated behavior and, consequently, light production.

While the bobtail *Sepiola*–*Vibrio* system exhibits a certain tolerance to thermal gradients due to the presence of symbionts adapted to both cold and warm waters, in other symbiotic systems, such as reef-building corals, thermal stress from even a 1 °C anomaly can result in mass bleaching and subsequent mortality [79,80]. In coral disease, this thermal limitation is exacerbated by a scenario of global warming. To overcome the accelerated breakdown of AHLs at elevated temperatures, coral pathogens must develop strategies to compensate for this loss by increasing the concentration of signals in the environment. The genus *Vibrio* has been found at high abundances in diseased corals compared to healthy corals [81,82,83]. Foundational work has demonstrated that *Vibrio* isolates from Black Band Disease increased the production of certain types of short- to medium- length AHLs, being 3-OH-C5-HSL the most commonly found, when temperatures reached 30 °C [28].

Recent studies have confirmed that this increase in QS-based pathogenic activity is one of the causes of coral mortality. For example, sole infection with *Vibrio fortis* was sufficient to cause coral bleaching and significantly reduced the photosynthetic activity of the algal symbiont. The algal population was barely affected, but the presence of *Vibrio fortis* produced an imbalance in the bacterial community, displacing strains with probiotic activity such as *Bacillus* and increasing the abundance of opportunistic pathogens like *Ralstonia* and *Burkholderia-Caballeronia-Paraburkholderia*. Furthermore, the authors found that applying enzymes with quorum quenching (QQ) activity had a positive effect on disease control, reinforcing the idea of QS playing a central role in coral pathogenicity [83]. A clear example of this temperature-dependent pathogenicity is *Vibrio coralliilyticus*, which utilizes a QS system driven primarily by AI-2 to regulate the expression of its virulence factors, such as Type VI secretion systems (T6SS) and proteases, deployed to attack the host during thermal stress events [84]. The primary gene responsible for AI-2 synthesis, *luxS*, was detected at a 3.7- to 4.0-fold higher abundance in bleached corals. Crucially, this pathway has been shown to contribute directly to coral bleaching by shifting the microbiome, altering the ratio of probiotic to pathogenic bacteria in a way that actively promotes the energy metabolism, chemotaxis, and biofilm formation of opportunistic pathogens [85].

In cold-water fish pathogens, both *Vibrio salmonicida* and *Vibrio wodanis* show that QS is strongly affected by temperature through changes in AHL production. In *V. salmonicida*, the LuxI/AinS system produces multiple AHLs (3-oxo-C6-HSL, 3-oxo-C8-HSL, C6-HSL, 3-OH-C10-HSL, C4-HSL, 3-oxo-C4-HSL) (Table 1), and its maximum concentration is between 6 °C and 12 °C, dropping drastically to 5% of the values at 6 °C when the bacteria are cultured at 16 °C [27]. This pattern corresponds with the optimal temperature at which cold-water vibriosis develops at 10 °C [86,87], suggesting that QS communication is enhanced in the thermal window of the disease. Something similar happens with the pathogen *V. wodanis*, responsible for ‘winter ulcer’ in salmon (*Salmo salar*) [88]. In this pathogen, the production of the AHL 3-OH-C10-HSL by the AinS synthase depends on cell density and is magnified at 6 °C [89]. These studies show how low temperature not only intervenes in the optimal growth of the bacteria but also in the increase in the production of AHL signals.

In microbial pathogens of warm-blooded animals, the effect of temperature on the QS signal could be illustrated with the case of *Yersinia pestis*, the bacteria responsible for the plague. This pathogen has a life cycle that alternates between different hosts, the flea vector, which exposes the bacteria to a colder environment (21 °C to 30 C), and the mammalian host at 37 °C. The QS system in *Y. pestis* depends on both AHLs (3-oxo-C6-HSL, C6-HSL and C8-HSL) (Table 1) and AI-2, which are produced both at 30 °C and 37 °C; however, the functional response to them is highly regulated by temperature. Both *aceA* and *aceB* participate in maltose fermentation and the glyoxylate bypass, and they respond to autoinducers at low temperature (21 °C to 30 °C) [30]. This cooler thermal window mimics the temperature of the flea gut, where bacteria have to persist during prolonged periods of time on limited, sporadic, nutrient-limited blood meals. This existing link between the QS response and the expression of genes related to carbon metabolism ensures the survival of the bacteria in the flea vector. On the other hand, when *Y. pestis* is found in the mammalian host, these responses are downregulated. This demonstrates that in *Y. pestis*, while autoinducer molecules are present, transcriptional output depends largely on the thermal landscape.

### 2.3. Fluid Flow and Viscosity

Beyond the chemical factors discussed above, whether autoinducers accumulate near the cells producing them or are carried away before reaching the receptor depends on the physical conditions surrounding the population, ranging from a viscous medium that favors retention to a more fluid one that washes them away. Because autoinducers are released into the extracellular space, they are continuously exposed to advection, the bulk movement of fluid that is a conserved feature of bacterial habitats, from the gut lumen and airway to rivers and the open ocean [17]. In this way, a cell senses the amount of autoinducer not only in terms of how much it can produce but also how quickly the signal is removed by fluid flow; a higher cell concentration is therefore required to reach the quorum under flow than in a static, well-mixed environment [10]. In addition, flow also promotes an uneven distribution of the QS signal across the population. In microfluidic channels that simulate confined host geometries such as the intestine, QS is repressed near the inlet, where advection removes the signal, whereas it is strongly activated near the end of the channel [90]. The same population can therefore have its QS switched off upstream and switched on downstream, so that position within the flow field, rather than cell density alone, sets the local response.

If we broaden the scope further and consider the effect of flow not on an isolated population but on a bacterial community, the molecules that different species share can shape the structure of that community. In an anaerobic microfluidic model of the gut using the human commensals *Bacteroides thetaiotaomicron* and *Bacteroides fragilis*, flow transported the metabolic by-products of the fermentation of polysaccharide dextran from the species that generate them to the species that need them to form a biofilm. In this way the population that needs the by-products to form the biofilm is situated downstream of the producer population. Furthermore, a sufficiently strong flow can abolish this cooperation by lowering the effective concentration of the shared good at the surface [91]. Flow therefore governs not only whether an autoinducer is kept or washed away but where in a bacterial community a QS-depending response can occur.

Another relevant factor that affects how bacterial populations and communities behave is viscosity. Beyond the *P. aeruginosa* work of Tang et al. [69], where acidic pH raises airway mucus viscosity, and Kümmerli et al. [92], where higher viscosity favors cooperation by limiting diffusion of a shared public good, the broader question of how matrix viscosity governs autoinducer retention remains largely unexplored. It is still unclear whether increased viscosity slows signal diffusion uniformly or creates local pockets of retention that let sub-quorum populations reach the threshold. Addressing this would require, for example, systematic measurements of autoinducer diffusion across defined viscosity gradients or comparisons of QS activation thresholds in host-mimicking matrices of varying viscosity such as mucus or biofilm exopolysaccharide. Clarifying how viscosity shapes signal retention would complete the picture of how the physical environment, alongside pH and temperature, governs whether a released signal ever reaches its receptor, and it would help explain QS behavior in the structured, often viscous microenvironments where host-associated bacteria actually live.

## 3. Environmental Effects on the Receptor and the QS Response

Environmental conditions not only control QS at the signal level, affecting its production and stability, but also strongly influence how receptors interpret the signal and convert it to a transcriptional response. Unlike the previous section, where we considered how temperature and pH affect different QS systems, this section will be organized around receptor architectures, describing how environmental factors affect each of them in specific ways.

### 3.1. Cytosolic LuxR-Type Receptors

**Figure 2:**
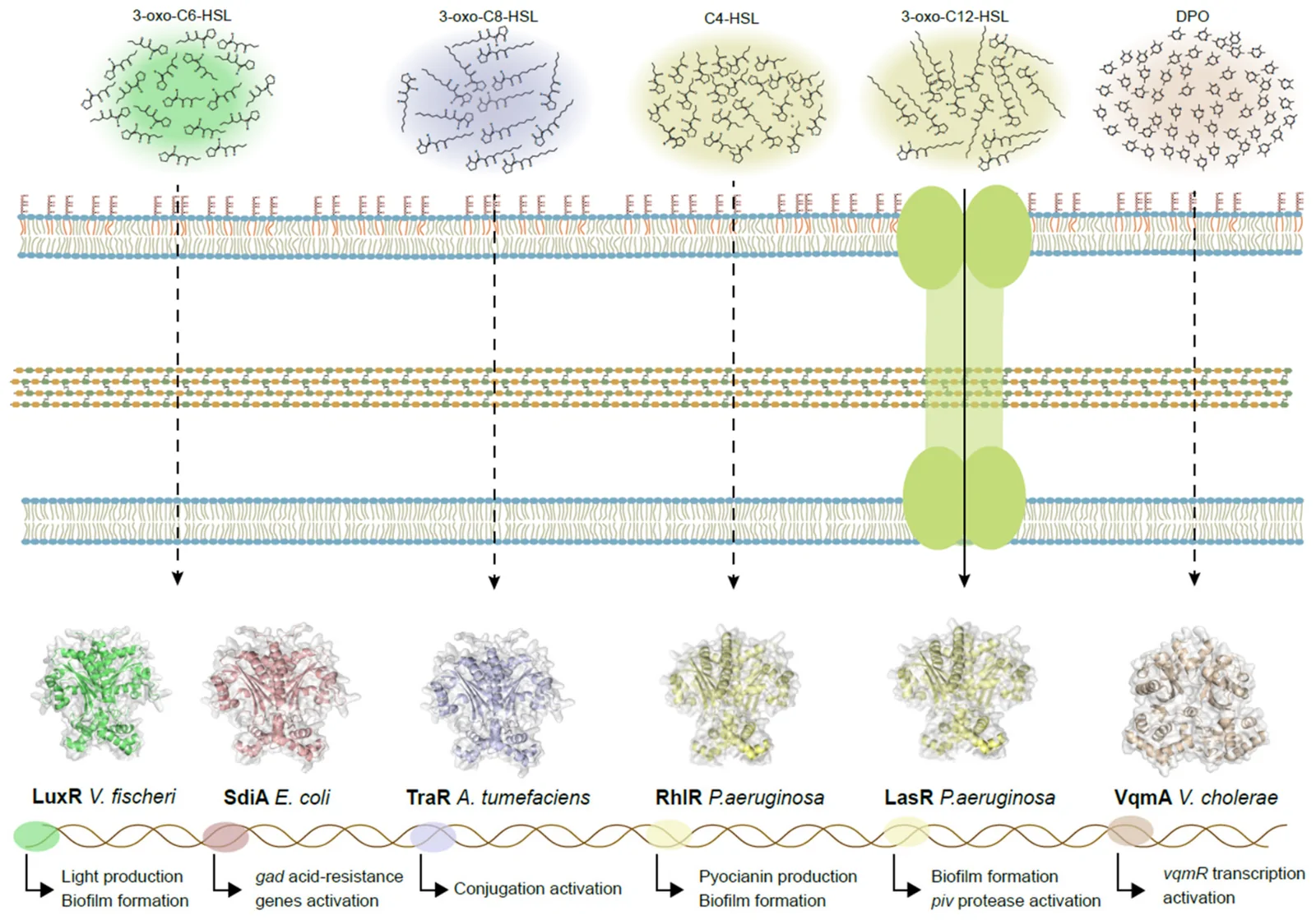
Signal transduction and phenotypic output of cytosolic LuxR-type and related QS receptors in Gram-negative bacteria. Extracellular autoinducers, including various *N*-acyl homoserine lactones (AHLs) and 3,5-dimethylpyrazin-2-ol (DPO), pass through the Gram-negative cell envelope either via passive diffusion (short-chain signals, dashed arrows) or active transport (long-chain signals, solid arrow). In the cytoplasm, these signals bind directly to their specific transcription factors. Upon activation, these cytosolic receptors (including classic LuxR-family proteins, the orphan receptor SdiA, and the DPO-receptor VqmA) bind to target DNA promoters to trigger diverse physiological responses, ranging from bioluminescence and conjugation to virulence factor production.

Cytosolic LuxR-type transcriptional proteins are predominantly found in Proteobacteria (Figure 2). They bind to AHLs following a stoichiometric ratio of typically 1:1, one AHL molecule to one protein molecule, in characterized canonical LuxR-AHL complexes [93]. Although many LuxR proteins have already been identified, to date there are only a few LuxR homologues that have been molecularly characterized, and these vary considerably in their amino acid sequence (~25% identity) and mode of action, although they share conserved regions in the DNA binding domain and in the AHL interaction domain [94,95,96].

Experimentally, the most extensively studied LuxR-type receptors behave as dimers that bind DNA only in the presence of a ligand, remaining as monomers in the cytoplasm in its absence [97]. Within the broad variety of LuxR-type homologs, there are systems in which autoinducer binding is irreversible, such as TraR with its 3-oxo-C8-HSL ligand in *Agrobacterium tumefaciens* for conjugation [31], or reversible, like LuxR and 3-oxo-C6 in *A. fischeri* for light production [29] (Figure 2). Other studies link ligand availability to receptor stability; for example, SdiA in *Escherichia coli* is relatively stable and folds correctly without exogenous AHL, but the presence of AHL increments the stability and the capacity to bind DNA [98], whereas LasR in *P. aeruginosa* binds AHL reversibly but is highly unstable in its absence (Figure 2) [99]. On the other hand, less well known are LuxR-type receptors that bind DNA as dimers in the absence of a ligand and dissociate upon autoinducer binding [100]. In most of these examples, except for SdiA, the bacteria also possess a cognate LuxI-type synthase, which produces the type of AHL recognized by its corresponding LuxR-type receptor, but many other Proteobacteria carry LuxR-type regulators that are not paired with a cognate LuxI-type synthase. Known as LuxR solos, they are able to detect endogenous and exogenous AHLs and non-AHL signals and are involved in intraspecies, interspecies, and interkingdom communication [101,102]. Importantly, SdiA detects AHLs produced by other commensal species in the bovine rumen, activating the *gad* acid-resistance genes and allowing *E. coli* to colonize the acidic stomach of the cow [103,104].

In *Vibrio alginolyticus*, temperature has a broader global effect in the sense that it not only alters gene expression patterns but also affects protein architecture and, consequently, how the protein recognizes its targets. First, as occurs in other species, organisms tend to modify gene expression networks to cope with thermal shock [105]. In species of *Vibrio*, heat shock (42 °C) causes proteolysis of QS master regulators, such as LuxR (*V. harveyi*), HapR (*Vibrio cholerae*), OpaR (*Vibrio parahaemolyticus*) and SmcR (*Vibrio vulnificus*) [106]. Earlier studies used chromatin immunoprecipitation and nucleotide sequencing (ChIP-seq) to measure the binding affinity of LuxR to all the promoters this protein interacts with across the genome at 30 °C, which is the optimal temperature for this bacterium, and in heat stress conditions, at 42 °C [107]. At 30 °C, 272 enriched loci were identified to carry LuxR-binding peaks, while at 42 °C this number was reduced to 22 enriched loci, which is consistent with the reduced expression of LuxR at 42 °C versus 30 °C. Analysis of the binding motif of LuxR demonstrated that this motif is shorter and diverges from canonical activator/repressor LuxR-type motifs at 42 °C, suggesting altered DNA recognition behavior under thermal stress. The affinity of LuxR to the virulence gene *asp* promoter was also reduced at 42 °C. Since *V. alginolyticus* is a major pathogen, this regulation balances the energetic cost of virulence factor expression against survival under thermal stress.

In *P. aeruginosa*, LasR and RhlR are two cytosolic LuxR-type QS receptors and are homologs of the *V. fischeri* receptor LuxR. In this bacterial system, it has been reported that one potential environmental input is the redox state of the cell and relies on cysteine residue 79 adjacent to the ligand-biding site of LasR [108,109] (Figure 2). In some bacteria, including *P. aeruginosa*, glutathione, a tripeptide thiol antioxidant, is one of the main compounds responsible for regulating the redox state of the cell. Glutathione, synthesized in two steps by GshA and GshB, is one of the main compounds setting the cell’s redox state. A *P. aeruginosa* mutant strain lacking the gene *gshA* demonstrated deficiency in biofilm formation, swarming, and pyocyanin production [110]. Further analysis demonstrated that a single mutation in *gshA* (causing a T44P substitution in GshA (GshA^T44P^)) that emerged during several passages of *P. aeruginosa* PAO1 in casein medium as a sole source of carbon and nitrogen produced a QS-hyperactive phenotype [111]. This evolved strain showed enhanced protease, pyocyanin, and C4-HSL production, where all of these increases were linked to QS-hyperactive behavior, presumably due to defective *gshA* that was responsible for lower levels of glutathione (normal behavior was restored by the addition of external glutathione). Surprisingly, the impact of the GshA^T44P^ might be to change the contributions of LasR and RhlR to QS gene activation, favoring RhlR, which is not a redox-active protein [108]. Interestingly, there may be additional mechanisms other than through the C79 residue of LasR, by which glutathione, and consequently the redox state of the cell, regulates QS gene activation. As an additional layer of QS control in PAO1, sulfane sulfur can modify LasR at several cysteines, and the modified protein activates transcription more effectively. The binding of LasR to its target DNA site is not affected by this modification, but it was significantly more effective than the unmodified version in activating transcription in both in vitro and in vivo assays [112]. Additionally. RhlR, as LasR, also participates in biofilm formation by activating the genes involved in rhamnolipid production and in pyocianin production [113] (Figure 2).

As we have seen previously, temperature is an environmental factor that not only affects signal stability but also strongly influences the receptor. In *P. aeruginosa* PAO1, protease IV (PIV), encoded by the *piv* gene, is a secreted serine protease and is considered a virulence factor. The expression of this gene does not change during exponential growth when the culture is exposed to different temperatures (25, 30, 37, and 42 °C). During the stationary phase, the *piv* gene is upregulated at 25 and 30 °C and downregulated at 42 °C. This transcriptional behavior is mirrored by mature PIV protein abundance. Further, reporter assays showed that the LasRI QS system is required for *piv* promoter activity at both 25 and 37 °C, but the absence of increased *lasR* transcript levels, LasR protein abundance, and 3-oxo-C12-HSL concentration at 25 °C suggests that enhanced *piv* expression at low temperatures is not driven by stronger global LasRI signaling (Figure 2). Two possible mechanisms for how temperature affects LasR regulation of *piv* may exist: direct regulation by LasR, binding stronger at 25 °C than at 37 °C to a putative *las* box upstream *piv* assisted by an unidentified *trans*-acting factor only present or active at 25 °C; or indirectly, by LasR activating an unidentified *trans*-acting factor, which in turn positively regulates *piv* more at 25 °C than at 27 °C [32]. Together, the results argue that thermoregulation of *piv* depends on promoter–LasR interaction, potentially via temperature-depending DNA properties or the intervention of an unidentified factor, rather than a global increase in QS signaling. This reinforces the idea that environmental conditions can reprogram QS responses at gene expression level.

The pathogen *V. cholerae* is able to thrive in a broad range of environments, from freshwater to salty oceans, fishes and crustaceans and from the stomach to the intestine in humans [114,115], creating the necessity of sensing the surroundings in a fast manner to develop a proper genetic response. In the human intestine in particular, this bacterium encounters two key stimuli: the absence of oxygen and the presence of host-produced bile salts [116,117]. One mechanism used by *V. cholerae* to integrate these environmental cues relies on the cytoplasmic receptor-transcription factor VqmA [118] (Figure 2). The autoinducer 3,5-dimethyl-pyrazin-2-ol (DPO) binds VqmA and activates expression of *vqmR*, which encodes the regulatory small RNA (sRNA) VqmR [119,120,121]. Unlike other LuxR-type cytosolic receptors, VqmA does not require DPO to activate *vqmR* transcription, although the autoinducer increases expression levels [119]. Notably, while DPO production increases under anaerobic conditions, another *V. cholerae* autoinducer, CAI-1, is not detected. Anaerobic conditions generally favor VqmA-dependent signaling, but when bile salts are present, they interfere with the complex VqmA-DPO by preventing formation of the C134-C134 intermolecular disulfide bond that enhances the so-called Holo-VqmA function. Consequently, *vqmR* expression decreases, relieving VqmR-mediated post-transcriptional repression of targets such as *tcpA* and *vpsL*, thereby increasing virulence and biofilm-associated gene expression and constituting another example of the redox state of the cell regulating QS-mediated responses. In this way, oxygen limitation and bile act like opposing inputs instead of redundantly, which allows *V. cholerae* to adjust the QS output according to intestinal microenvironmental conditions. Although the VqmA-DPO system illustrates how host-derived cues such as oxygen limitation and bile salts modulate quorum-sensing output during intestinal colonization, *V. cholerae* must also integrate physicochemical signals encountered outside the host. In this context, biofilm formation is crucial for survival in aquatic environments where it is exposed to fluctuations in temperature. At lower temperatures (15–25 °C), *V. cholerae* forms thicker and more structured biofilms than at 37 °C, a phenotype accompanied by increased intracellular c-di-GMP levels. This response depends on the combined action of multiple diguanylate cyclases (DGCs), four of which carry sensory input domains that could sense environmental inputs such as lower temperatures; a strain lacking them fails to elevate c-di-GMP after a shift from 37 °C to 15 °C, though the authors note that the transmembrane domains of these proteins may also contribute to sensing. Taken together, these observations show how *V. cholerae* can recognize host-associated cues to behave as a pathogen during its colonization phase and to respond to temperature changes during environmental persistence by modulating QS [122].

In all the examples above, the environment acts on the LuxR-type receptor protein itself, whether by altering its stability, modifying key residues, reshaping its DNA recognition, or controlling its proteolysis. A mutualistic case in the rhizosphere symbiont *Sinorhizobium meliloti* adds a further mode, in which the environment instead tunes how much receptor is produced. The LuxR-type receptor ExpR, together with the synthase SinI, controls the exopolysaccharide and motility programs that underlie nodulation of its *Medicago* host [123]. What is different here is that the environment does not act on the ExpR protein at all. Instead, soil- and host-derived cues repress *expR* transcription: L-canavanine from alfalfa exudates and water-soluble humic substances from the soil both lower *expR* expression, and in the case of humic substances this happens through QsrR (SMc03890), a DeoR-family repressor that binds the *expR* promoter directly. The result is less ExpR, less AHL, and reduced exopolysaccharide II production [124,125]. This dampening might seem counterproductive, but because the AHL–ExpR complex normally represses nitrogen-fixation genes, turning QS down actually improves symbiotic nitrogen fixation. In this mutualist, then, receptor levels are set by host and soil chemistry rather than by cell density alone.

### 3.2. Membrane Histidine-Kinase Receptors

**Figure 3:**
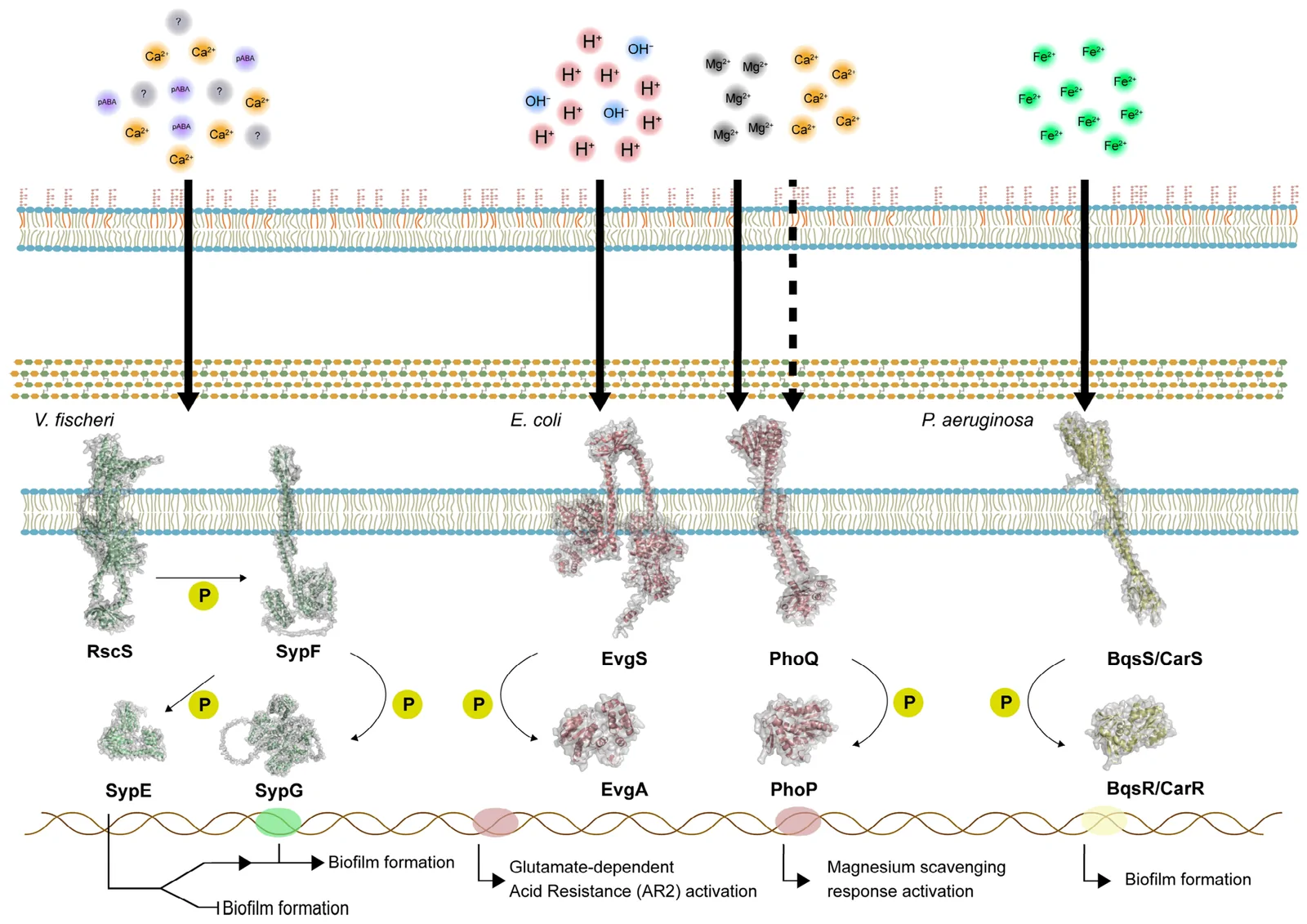
Transmembrane signal transduction and phosphorelay architectures of representative Gram-negative bacterial two-component systems (TCSs). The diagram maps the spatial organization of TCS pathways across three panels: *V. fischeri* (**left** panel), *E. coli* (**middle** panel), and *P. aeruginosa* (**right** panel). The upper section of each panel depicts the specific environmental stimuli (Ca^2+^, pABA, H^+^, OH^−^, Mg^2+^ and Fe^2+^) associated with each system. Solid black arrows trace the primary pathways of signal transduction from integral membrane sensor kinases to their respective cytosolic response regulators. Yellow circles denote phosphorylation (P) events. The dashed black arrow in the middle panel (*E. coli*) denotes a secondary, lower-affinity interaction between Ca^2+^ and the PhoQ sensor, contrasting with the primary Mg^2+^ signal. Downstream phenotypic outputs are indicated below the DNA binding sites.

Two-component signal (TCS) transduction systems are highly conserved among bacteria and archaea and are comprised of a membrane histidine-kinase (HK) receptor and its cognate response regulator (RR) [126]. When a signal is detected, HKs autophosphorylate their conserved histidine residues and subsequently a phosphotransfer reaction is performed to the aspartate-containing module of the RR. This RR, in most cases, acts as a transcriptional regulator, controlling the expression of genes involved in biofilm formation, virulence, and QS as a response to the external or the internal environment of the cell [127,128]. Symbiotic bacteria that frequently transition between free-living and host-associated lifestyles require mechanisms such as membrane histidine kinase systems to detect rapid environmental changes, including oxygen levels, osmolarity, nutrient availability, and host-derived stresses, while simultaneously sensing local population density through QS to act coordinately producing any output (bioluminescence, biofilm formation, or virulence factor production). For example, in the squid symbiont *V. fischeri*, host colonization and biofilm development are controlled by a complex two-component phosphorelay involving the hybrid sensor kinase RscS, which has been demonstrated to respond in culture to *para*-aminobenzoic acid and calcium [129], as well as the response regulators SypE and SypG (Figure 3). A key finding is that SypF functions downstream of RscS to control these regulators, but its kinase activity is not required in vivo; instead, only the non-enzymatic HPt domain is essential, enabling a bypass of canonical signaling and illustrating the plasticity of TCS regulatory architectures [130].

Another relevant example of environmental control where a two-component system is involved can be found in the gut, where pH and osmotic fluctuations play important roles, as previously shown. In *E. coli*, the EvgAS system is activated under acid stress, moderated at pH = 5.5 and increased at pH = 2, promoting resistance to acid genetic programs (including AR2 regulon), although available evidence suggests that EvgAS output is context-dependent and modulated by additional inputs rather than by pH alone [131] (Figure 3). This integration is also supported by interaction or cross-regulation with other types of stress-sensing two-component systems, such as PhoPQ, which senses metal (primarily Mg^2+^) and to a lesser extent Ca^2+^, via an apparatus encoded by the *phoPQ* operon that acts in tandem with EvgAS [132]. Additionally, to complicate it even more, it seems that indole inhibits the EvgAS system, where the AR2 promoter is downregulated, subsequently silencing the stress response in *E. coli* [131]. Indole appears to counteract EvgAS signaling through two complementary mechanisms: it can modulate intracellular pH, and it also functions as a bacterial communication signal that reshapes phenotypes linked to persistence and infection. In this context, suppression of a costly stress-response program when neighboring bacterial density is high may help *E. coli* allocate resources more efficiently and optimize growth under complex environmental conditions [133].

**Figure 4:**
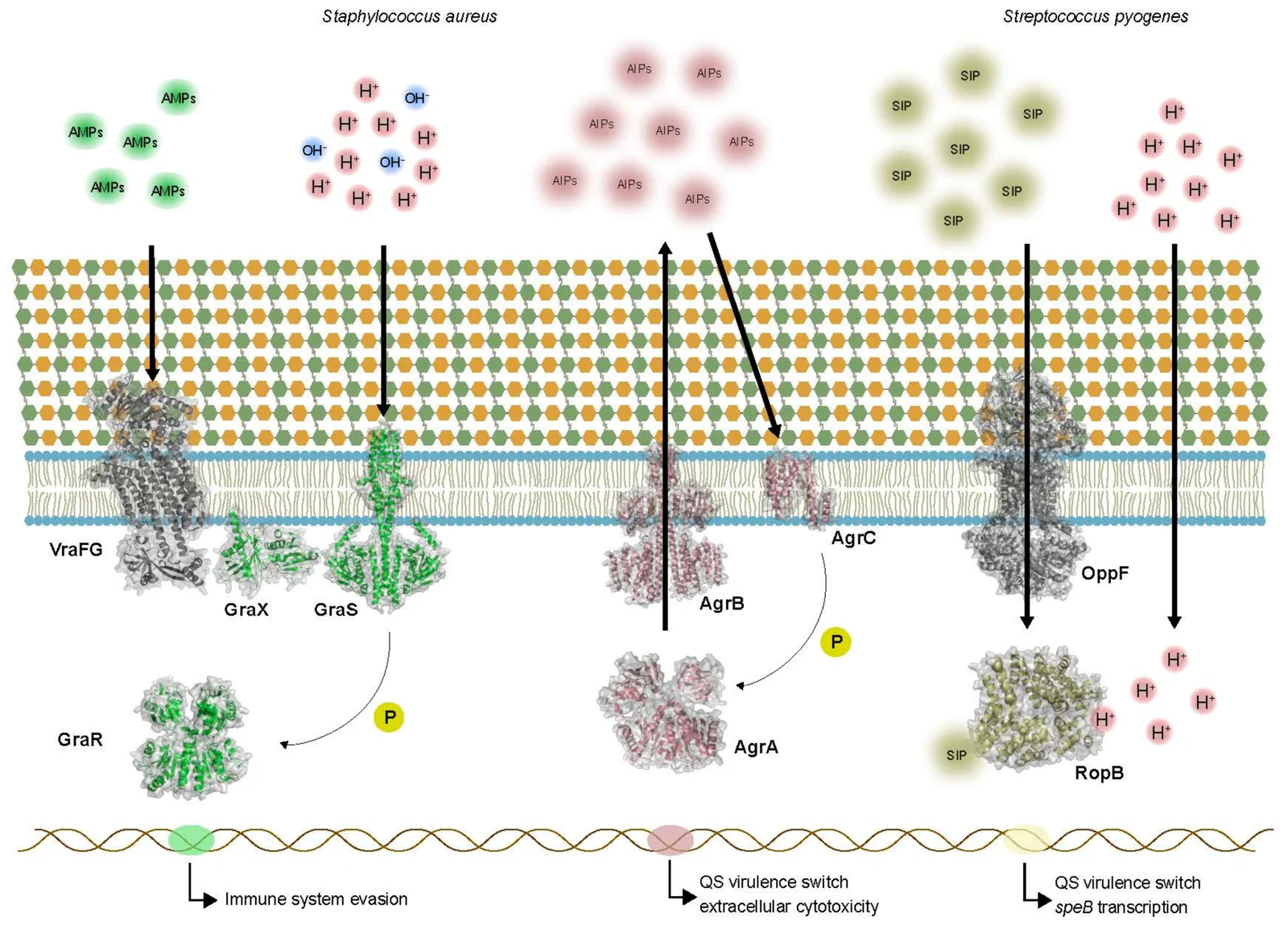
Signal transduction of environmental cues in Gram-positive bacteria. The diagram maps the spatial organization of sensory and QS pathways in *Staphylococcus aureus* (**left** and **middle**) and *Streptococcus pyogenes* (**right**). Top panels depict the specific environmental stimuli (AMPs, H^+^, OH^−^, AIPs, and SIPs) associated with each system. Solid black arrows show the primary route of each element, from the outside to the protein it interacts with or it is detected by. The solid black arrow pointing towards the exterior of the cell denotes the active export of AIPs by AgrB. Yellow circles denote phosphorylation (P) events. Downstream phenotypic outputs are indicated below the DNA binding sites.

The environmental pH can function not only as a modulator of the signal detection in bacteria but also as an adaptation driver. During macrophage infection, *Staphylococcus aureus* initiates replication within intact, acidified phagolysosomes (luminal pH approximately 5.4). To proliferate in this cellular compartment, *S. aureus* requires GraXRS, which is a special type of histidine kinase response regulator with an extra component, a helper membrane-associated protein (GraX) that assists tandem GraS-GraR mediated signaling (Figure 4). Importantly, this system acts in tandem with the ABC transporter VraFG. While GraS senses low pH and participates in the activation of genes implied in acidic environment protection, VraFG functions as a co-sensor that directly detects positively charged host antimicrobial peptides (AMPs) like defensins. This integrated signaling drives the expression of effectors such as *mprF*, which functions as an antimicrobial protective that renders the outer membrane less negatively charged, preventing the binding of these AMPs. Alternatively, other genetic programs required for extracellular cytotoxic behavior, like the *agr* QS regulator or the two-component system SaeRS, are not essential for *S. aureus* to multiply in the phagolysosome at early infection stages. Together, these findings highlight the role of environment signals (pH in this example), unifying QS and host–pathogen interactions and determining the transition between intracellular survival and proliferation at early infection stages [47,134].

Apart from pH, temperature, or cell density, other factors such as metallic ions can participate in QS regulation. *P. aeruginosa* has the membrane HK BqsS/CarS, which is a direct and selective sensor of environmental Fe^2+^ (Figure 3). Earlier studies purified the homodimer state of BqsS and demonstrated that it binds a single Fe^2+^ ion within its periplasmatic domain containing an N/O-rich ligation sphere that includes Glu^48^ as a key metal ligand [135]. This binding strongly increases the ATPase activity of BqsS, whereas Fe^3+^, Ca^2+^, and other tested metals do not produce the same effect. Mutational analysis separated metal binding from signal transduction, identifying a residue required for signaling rather than Fe^2+^ coordination. These results establish a direct transmembrane signaling model triggered by Fe^2+^ which affects *P. aeruginosa* social behavior, increasing the importance of HKs in host–pathogen interaction.

### 3.3. Intracellular Peptide Receptors

In Gram-positive bacteria, AIPs can be detected by membrane-associated signal transduction proteins, as was described in the introduction of this review with the AgrC system of *S. aureus*, or by receptors located in the cytoplasm. In membrane-associated proteins, the surrounding environment interacts more directly with the signal receptor, through changes in pH, redox potential, or nutrient availability (Figure 4). In comparison, AIPs detected by cytosolic factors have a series of control layers—production, modification, secretion, exposure to the environment, and import—before finally interacting with the cytosolic receptor. This complex control network allows the bacterium to adapt its collective behavior and optimize the output long before the signal; in this case, AIPs physically contact the receptor. In this section, we will focus on this AIP–cytosolic receptor interaction and on how it can be affected by the environment. The RRNPP family (Rap, Rgg, NprR, PlcR, PrgX) is a major group of proteins that participate in intracellular peptide detection in Gram-positive bacteria [136].

A good example of environmental pH and peptide signaling controlling virulence is the production of the QS-regulated virulence factor SpeB by the pathogen *Streptococcus pyogenes* (Figure 4). In this bacterium, the short oligopeptide autoinducer SIP is the population density signal, and the cytosolic transcriptional factor RopB is the intracellular regulator. Because RopB resides in the cytosol, it relies on the transmembrane ABC transporter OppF to act as an importer, actively internalizing the extracellular SIP signal. The main readout is SpeB, a secreted cysteine protease whose expression is highest at acidic pH, and its activation/activity are also better in acidic conditions [137]. Environmental acidification does not primarily affect SIP uptake mediated by OppF but rather modulates SIP recognition by RopB through a pH-sensitive histidine switch (H144) located at the base of the SIP-binding pocket [138]. At mild acidic pH (5.5–6), protonation of H144 stabilizes an intramolecular interaction network involving Y176, Y182′, and E185′, which increases RopB-SIP binding affinity and promotes strong *speB* transcription. This demonstrates that high SIP and low pH yield maximal virulence output, helping *S. pyogenes* avoid premature expression of energetically costly virulence factors in neutral pH (not adequate for protease activity). The in vivo data strongly reinforce this ecological interpretation: low-pH-preconditioned inoculum produces earlier and more severe tissue lesions, whereas mutations that impair SIP-RopB binding or disrupt the H144 switch strongly reduce pathogenicity. Thus, this host-associated acidification is not a “meaningless” stress for the bacterium but an important cue that affects and reshapes the QS receptor state and determines disease severity.

## 4. Limitations and Future Perspectives

Any synthesis of environmental QS modulation is currently constrained by the historical trajectory of the field: the literature remains heavily skewed toward Gram-negative, AHL-mediated systems. Consequently, the environmental factors affecting Gram-positive and archaeal QS networks are significantly underrepresented. Moreover, while host-associated environments like the mammalian gut represent hot spots of microbial crosstalk, detailed mechanistic studies of QS modulation in these habitats remain sparse. Bridging this gap requires a methodological shift. Future efforts must prioritize in situ signal quantification. Mass spectrometry imaging (e.g., MALDI-MSI) can map autoinducer gradients directly across host tissues, resolving where signals accumulate or are depleted at spatial scales relevant to colonization, rather than inferring concentrations from bulk homogenized samples. Because this approach preserves spatial information, it can distinguish, for example, whether a signal is retained within host mucus or cleared from it, a distinction central to the pH and viscosity effects discussed above. Spatial transcriptomics (e.g., seqFISH or par-seqFISH) complements this by reporting the cellular response rather than the signal itself: tagging QS-related mRNA transcripts with fluorescent barcodes reveals which cells within a gut or coral microbiome are actively responding while preserving native spatial architecture. Combining the two, mapping signal distribution and transcriptional response in the same tissue, would link the physicochemical state of a host niche to the QS output it produces, closing the gap between in vitro mechanism and in vivo behavior.

These gaps matter for the symbiotic systems that motivate this review. In both mutualistic and pathogenic associations, the outcome, whether stable colonization, nutrient exchange, or virulence, depends on QS decisions made within specific host microenvironments that bulk methods cannot resolve. Mapping QS in situ is therefore needed to understand how environmental context shapes the establishment and maintenance of host–microbe symbioses.

## 5. Conclusions

QS should no longer be understood only as a mechanism by which bacteria estimate their own population density. Across the examples discussed in this review, QS is better described as an environmentally contextualized regulatory process, where the final output depends on the interaction between signal production, signal persistence and transport, receptor state, and the physicochemical conditions of the surrounding environment. The same signaling molecule can carry a different biological meaning depending on whether it is produced in an alkaline or acidic environment, under low or high temperature, in a biofilm, in host mucus, or under strong host-derived stress. Both autoinducer signals and the proteins that detect them are modified by the environment, so QS ceases to be a static process and becomes a dynamic one, in which multiple layers of control shape the final output. This perspective is especially important in host-associated systems, where bacterial success depends on integrating population-level communication with the rapidly changing conditions of the host. During symbiosis, such integration can support stable colonization, nutrient exchange, and coordination between partners, while in pathogenesis it can determine the timing, intensity, and spatial restriction of virulence factor production. Understanding QS as an environmentally tuned process therefore offers a more realistic account of microbial behavior than models based on cell density alone. This view also has practical consequences: if the environment determines when and how strongly QS activates, then anti-virulence therapies and efforts to engineer stable microbial communities must account for the pH, temperature, flow, and viscosity of the specific host niche, rather than relying on standard laboratory conditions. Three points follow from this review. First, the meaning of a QS signal depends on the signal, the receptor, and the environment together, not on cell density alone. Second, a given environmental factor can drive QS toward opposite outcomes in different systems. Third, QS behavior measured in vitro cannot be assumed to hold in vivo without accounting for the physical and chemical conditions of the host niche.

## Figures and Tables

**Table 1:** Structural diversity of acyl-homoserine lactone (AHL) autoinducers. Chemical structures and standard nomenclature of representative AHLs utilized by Gram-negative bacteria in QS.

Structure	Name	Species	Reference
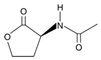	C2-HSL	*Gluconacetobacter*	[20]
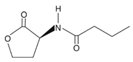	C4-HSL	*Vibrio salmonicida* *Vibrio wodanis*	[27]
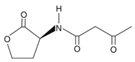	3-oxo-C4-HSL	*Vibrio harveyi*	[27]
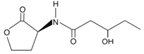	3-OH-C5-HSL	*Vibrio harveyi*	[28]
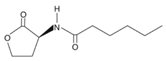	C6-HSL	*Vibrio salmonicida* *Vibrio wodanis*	[27]
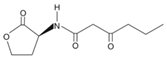	3-oxo-C6-HSL	*Vibrio salmonicida* *Vibrio wodanis* *Vibrio fischeri*	[27,29]
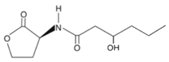	3-OH-C6-HSL	*Pseudomonas chlororaphis*	[26]
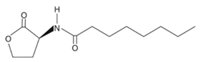	C8-HSL	*Yersinia pestis*	[30]
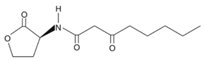	3-oxo-C8-HSL	*Agrobacterium tumefaciens* *Vibrio salmonicida* *Vibrio wodanis*	[27,31]
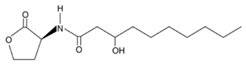	3-OH-C10-HSL	*Vibrio salmonicida* *Vibrio wodanis*	[27]
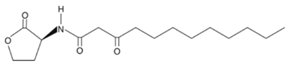	3-oxo-C12-HSL	*Pseudomonas aeruginosa*	[32]
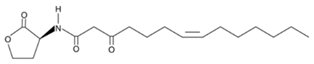	3-oxo-C14:1-HSL	*Vibrio* sp.	[24]
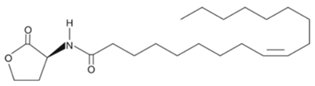	C18:1-HSL	*Dinoroseobacter shibae*	[22]
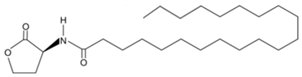	C20-HSL	*Rhodovulum sulfidophilum*	[21]

## Data Availability

No new data were created or analyzed in this study. Data sharing is not applicable to this article.

## Abbreviations

AAH	N-acylamino acid hydrolase
*agr*	Accessory gene regulator
AHL	*N*-acyl homoserine lactone
AI-2	Autoinducer-2
AIP	Autoinducing peptide
AMP	Antimicrobial peptide
ASL	Airway surface liquid
BBD	Black band disease
CF	Cystic fibrosis
CFTR	Cystic fibrosis transmembrane conductance regulator
DGC	Diguanylate cyclase
DPD	4,5-dihydroxy-2,3-pentanedione
DPO	3,5-dimethyl-pyrazin-2-ol
HK	Histidine kinase
LC-MS	Liquid chromatography–mass spectrometry
PIV	Protease IV
QQ	Quorum quenching
QS	Quorum sensing
RR	Response regulator
SIP	Short oligopeptide autoinducer
SML	Surface mucopolysaccharide layer
sRNA	Small RNA
T6SS	Type VI secretion system
TCS	Two-component signal/two-component system
